# Hypospadias and Increased Risk for Psychiatric Symptoms in Both Childhood and Adolescence: A Literature Review

**DOI:** 10.3389/fpsyt.2022.799335

**Published:** 2022-02-23

**Authors:** Tingting Jin, Weizhou Wu, Maolei Shen, Haiya Feng, Ya Wang, Shixiong Liu, Xin Li, Shankun Zhao

**Affiliations:** ^1^Department of Urology, Taizhou Central Hospital (Taizhou University Hospital), Taizhou, China; ^2^Department of Urology, Maoming People's Hospital, Maoming, China

**Keywords:** hypospadias, psychiatric disorder, risk, comprehensive review, depression

## Abstract

Hypospadias is one of the most common congenital malformations in boys. Due to abnormal appearance in the penis with abnormal urination and erection, patients with hypospadias were vulnerable to suffering from stress and psychiatric difficulties. The present study aims to summarize all the current evidence of the association between hypospadias and the risk of psychiatric disorders by a comprehensive review. Seventeen clinical studies were identified in the four electronic databases. A total of 953,872 participants were involved, while 15,729 of them were hypospadiac patients and the remaining 938,143 were normal controls. The standard age for surgery for hypospadias ranged from 20.4 months to 21.5 years. Eight out of seventeen (8/17, 47%) included studies explicitly showed that patients with hypospadias had a significantly higher risk of psychosocial disorders (all *P* < 0.05). Specific types of psychiatric disorders included depression, anxiety, shyness, timidness, isolation, fear of ridicule, attention-deficit hyperactivity, autism spectrum, behavioral/emotional disorders, temper tantrums, emotionality, affective, psychosexual problems, and suicidal tendencies. Based on this review, psychiatric illnesses are frequently detected in hypospadiac patients' childhood, thus proper psychiatric guidance and early interventions from physicians, nurses, and parents may help these children to grow into less affected men.

## Introduction

Hypospadias, one of the common diverse urologic anomalies among children, is characterized by a failure of urethral groove closure leading to an opening on the ventral aspect of the penis (1, 2). The severity of hypospadias depends on the failure timing of the incomplete fusion of the urethral folds. In hypospadias children, the urethra opens ventrally anywhere from the glans to as far back as the perineum, while distal hypospadias accounts for the majority of cases (3). Hypospadias occurs in 2–43 out of 10,000 live male births and exists along a spectrum of severity (4). The exact etiologies of hypospadias are still unclear, it is believed that genetic factors, endocrine hormones (i.e., androgens), environmental components contribute to the pathogenesis (5). There is no consensus on the timing of surgery (cognitive factors, risk of anesthesia, surgical considerations, psychological, developmental, and psychosexual considerations). Based on expert opinion, surgery is recommended between 6 and 18 months of age (6).

At present, debate persists whether hypospadias *per se* or surgical procedures have a negative influence on later psychiatric development. Mounting studies (7, 8) have examined psychiatric development and psychiatric symptomology in patients with hypospadias. Though a trend toward a potential association between hypospadias and psychiatric symptoms, the evidence has been controversial (9). The present study aims to summarize the published data related to this issue. Based on this review, it may be instructive to help the clinicians being conscious of the hazardous effect of hypospadias in the development of psychiatric disorders. Furthermore, it is meaningful to take some psychointervention from parents, doctors, and nurses to alleviate psychiatric ailments for the sufferers.

## Methods

To identify the eligible studies focusing on the relationship between hypospadias and psychiatric disorders, four electronic databases including MEDLINE, EMBASE, Cochrane Library, and PsychINFO were systemically retrieved up to date to August 01, 2021. The searching strategy used for screening the qualified publications in MEDLINE by the MeSH and terms was: ((“Hypospadias”[Mesh]) OR (Hypospadia)) AND (((((((((((((((((((((((((“Mental Disorders”[Mesh]) OR (Psychiatric disorder)) OR (Mental Disorder)) OR (Behavior Disorders)) OR (Depressions)) OR (Depressive Symptoms)) OR (Depressive Symptom)) OR (Symptom, Depressive)) OR (Symptoms, Depressive)) OR (Emotional Depression)) OR (Depression, Emotional)) OR (Depressions, Emotional)) OR (Emotional Depressions)) OR (Angst)) OR (Nervousness)) OR (Hypervigilance)) OR (Anxiousness)) OR (Social Anxiety)) OR (Anxieties, Social)) OR (Anxiety, Social)) OR (Social Anxieties)) OR (Stress)) OR (psychology)) OR (psychological)) OR (psychiatry)). Furthermore, we also reviewed the reference list to detect additional studies by a manual inspection. Duplicated data, review articles, letters/comments, case reports, meeting abstracts, and animal experiments were excluded in this study.

The process of study selection was conducted by two authors independently. Any ambiguities were resolved by the corresponding author. A standardized data collection table was used to extract the important data (e.g., the names of the first author, geographical distribution of the study, study design, publication year, mean age of the patients, the number of participants from the study group and the control group, the descriptions of psychological disorders in every single study, the age at criterion operation of hypospadias, and the assessment of psychiatric symptoms).

## Results

### Literature Search and Eligible Study Characteristics

**Figure 1:**
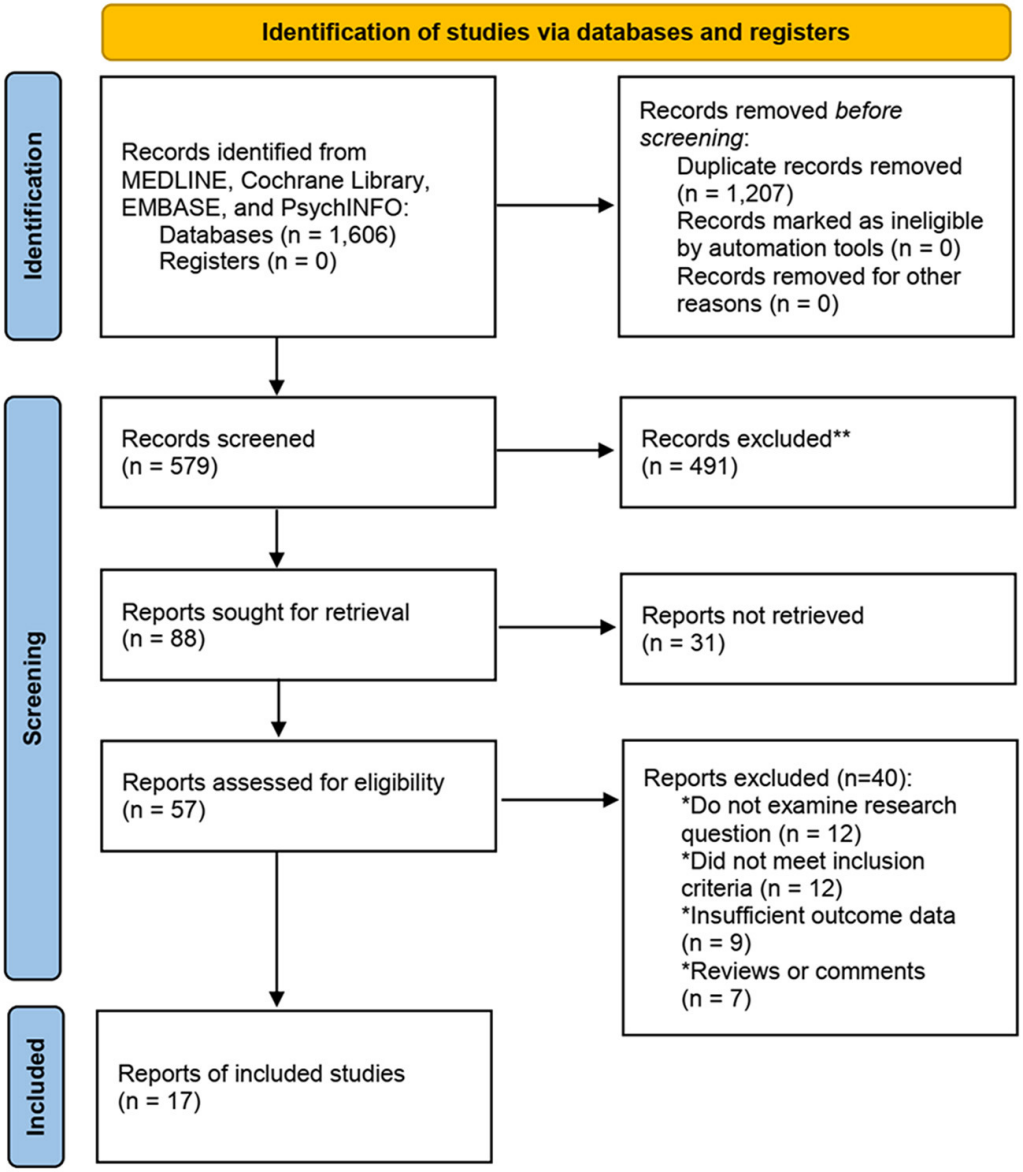
Flow chart of study selection.

Figure 1 showed the search flowchart for screening eligible studies related to the topic of hypospadias and psychiatric disorders. During the initial search, 1,606 records were identified, of which 567 were from MEDLINE, 411 from EMBASE, 347 from Cochrane Library, and 281 from PsychINFO database. Finally, 17 eligible clinical studies (3, 10–25) containing with data met our predefined inclusion criteria. Six of these studies (13/17, 76%) had provided the data from both the hypospadias and the healthy control group. A total of 953,872 participants were involved, while 15,729 were hypospadiac patients and 938,143 were normal controls. Among the 17 studies included, eight were case-control designed, five for cohort designed, one for cross-sectional designed, one for randomized controlled trial (RCT), and one for prospective designed. The publication years of these selected studies ranged from 1982 to 2021.

The mean age of the hypospadiac patients ranged from 20.4 months to 34.2 years. The standard surgical age for hypospadias ranged from 20.4 months to 21.5 years. Types of psychiatric disorders reported in the 17 eligible studies included depression, anxiety, shyness, timidness, isolation, fear of ridicule, attention-deficit hyperactivity, autism spectrum, behavioral/emotional disorders, temper tantrums, emotionality, lack of vitality, esteem troubles, affective disorder, gender identity, psychosexual problem, gender-role behavior, and suicidal tendencies. The assessments of these psychiatric disorders among hypospadiac patients included the following methods, e.g., an interview by the psychiatrists or nurse specialists, child psychiatric symptoms list, standardized intelligence test, the Rorschach test, Spielberger anxiety questionnaire, Goldberg General Health Questionnaire, Beck Depression Inventory (BDI), Self-rating Depression Scale (SDS), Self-rating Anxiety Scale (SAS), Children's Fear Scale, Post-Hospitalization Behavioral Questionnaire, Modified Yale preoperative anxiety scale, Autism spectrum disorders, Attention deficit hyperactivity disorder, Behavioral/emotional disorders, Body-Esteem Scale for Adolescents and Adults; Psychological General Well-Being Index, Genital Examination Distress Scale, Ages and Stages Questionnaire and ASQ-Social Emotional Scale, Psychosexual milestones, Gender-Role Questionnaire, and the Mini International Neuropsychiatric Interview. The characteristics of the 17 included studies were summarized in Table 1. We had broken the hypospadias patients up by age with childhood, adolescence, and adulthood.

**Table 1:** Characteristics of the 17 included studies.

**Study**	**Study design**	**Mean age (years)**	**Number of patients**	**Types of psychiatric disorders**	**Descriptions of psychiatric disorder**	**Age at criterion operation of hypospadias (years)**	**Assessment of psychiatric disorder**
**Childhood**							
Sanders (3) UK	RCT	20.4 months	20	Distress	NA	Mean: 20.4 months	Urology nurse specialist
Turk et al. (22) Turkey	Prospective study	5–12	30	Fear and anxiety	Imaging of micturition at home by using a video camera for outpatient visits following hypospadias surgery decrease the fear and anxiety of children.	5–12 years	The Children's Fear Scale
Duarsa et al. (15) Indonesia	Case-control	S: 5.9 ± 3.9 C: 5.5 ± 2.5	S: 10 C: 19	Distress	Poor Genital Examination Distress Scale score was detected more in the hypospadias group compared to the control group, but this was not statistically significant (30 vs. 15.8%, OR = 2.28, *P* = 0.331).	NA	Genital Examination Distress Scale
Luo et al. (16) China	Cohort	2–12	177	Temper tantrums, emotionality	Temperament emotionality: OR = 1.112 (1.011, 1.224), *P* = 0.029; Emotionality: OR = 1.148 (1.049–1.256), *P* = 0.001; there is an association between child anxiety upon entrance into the operation room and negative postoperative behavioral changes but not in the adjusted regression analysis.	2–12 years	Post-Hospitalization Behavioral Questionnaire and Modified yale preoperative anxiety scale
**Adolescence**							
Schonbucher et al. (20) Switzerland	Cross-sectional	S: 10.8 ± 3.2 C: 11.1 ± 3.2	S: 68 C: 68	Gender-role behavior	Patients with hypospadias did not significantly differ from the control subjects with regard to gender-role behavior. Gender-role behavior was significantly negatively correlated to the patients' age at last surgery and positively associated with follow-up since last surgery (*P* < 0.05).	Mean: 3.2 ± 2.5 years	Gender-Role Questionnaire
Butwicka et al. (13) Sweden	Case-control	13.2	S: 9,262 C: 463,100	Attention-deficit hyperactivity, autism spectrum, and behavioral/emotional disorders	The lifetime prevalence of any psychiatric disorders was 9.7% for cases with hypospadias and 7.6% for controls (OR 1.3, 95% CI: 1.2–1.4).	NA	Information on psychiatric disorders was extracted from the National Patient Register in Sweden
**Adulthood**							
Berg et al. (11) Sweden	Case-control	S: 27.2 C: 26.9	S: 34 C: 36	Depression, anxiety, shyness, timid, isolated	Hypospadias is associated with more psychiatric symptoms than controls in adult age (*P* < 0.05); The most marked difference is depressiveness (15/34 in hypospadias group and 5/36 in the control); more stress reactions are found in the hypospadias patients (*P* < 0.01).	3.0–9.5 years	An extensive interview was performed with each subject by an experienced psychiatrist; child psychiatric symptoms list
Berg and Berg (12) Sweden	Case-control	20–35	S: 33 C: 36	Depression, anxiety, shyness, timid, isolated	Patients with hypospadias have significant higher risk of hostility, reduction of social relations and emotional relations, anxiety, less self-esteem, and less activity (*P* < 0.05); but no significant differences on depression and authoritarian submission.	3.0–9.5, Mean: 5.6 years	A psychological test battery, including standardized intelligence test, the Rorschach test
Miller and Grant (17) UK	Cross-sectional	17.7–36.6	19	Depression	Four (4/19) patients reported marked impairment of psychological well-being.	2–8; median: 3 years	Spielberger Anxiety Questionnaire, Goldberg General Health Questionnaire, and BDI
Wang et al. (24) China	Case-control	S: 24.3–28.4 C: 24–35	S: 130 C: 50	Depression, anxiety, fear of ridicule	Depression and anxiety were significantly higher in the hypospadias group than in the control group (*P* < 0.001); the incidence of depression and anxiety were significant differ between groups A and B (*P* < 0.01), groups A and C (*P* < 0.01).	Median: Group A: 6.5, Group B: 13.0 Group C: 21.5	SDS, SAS
Schlomer et al. (19) USA	Case–control	S: 34 C: 33	S: 736 C: 684	Psychosexual problems	The mean number of mentally unhealthy days was significantly differed between severe untreated hypospadias and the controls (12.1 vs. 6.5 days, *P* = 0.017).	Untreated hypospadias (No surgery)	Psychosexual milestones
Ortqvist et al. (34) Sweden	Cohort	S: 34 C: 33	S: 167 C: 169	Core gender identity and gender role behavior	There was no association with core gender identity and gender role behavior between hypospadias patients and controls. However, patients with proximal hypospadias had a higher gender identity score (*P* = 0.02) and gender role behavior score (*P* = 0.04) compared with men with distal hypospadias.	Mean: 5 ± 4 years	A 12-item questionnaire
Andersson et al. (10) Sweden	Cohort	S: 14–35 C: 15–29	S: 64 C: 25	Anxiety, depressed mood, positive well-being, self-control, vitality, and esteem	No differences in anxiety, depressed mood, positive well-being, self-control, and vitality between hypospadias and control group (all *P* > 0.05).	NA	Body-Esteem Scale for Adolescents and Adults; Psychological General Well-Being Index
Ortqvist et al. (18) Sweden	Cohort	S: 34.2 ± 7.1 C: 27.2 ± 6.7	S: 33 C: 47	Affective, suicidal, and anxiety symptoms were more common in hypospadias patients than the controls, but this did not reach statistical significance	Psychiatric symptoms did not differ significantly between the hypospadias and control group, as well as between different severity or phenotype groups.	Median: 4 years	Mini International Neuropsychiatric Interview
**No age information or the great age variation**			
Wang et al. (23) China	Case-control	S: 3–26 C: 24–35	S: 130 C: 50	Depression and anxiety	Hypospadiac patients have significantly higher occurrences of depression/anxiety than the normal controls (*P* < 0.001); Also, the postoperative SDS/SAS scores were higher in patients with hypospadias; Patients with proximal hypospadias and multiple procedures have higher risk of sexual psychological problems.	Median: 16.5 years	SDS, SAS
Skarin et al. (21) Sweden	Cohort	NA	S: 4,738 C: 473,800	Autism, behavioral/emotional disorders, and attention deficit hyperactivity disorder	Patients with hypospadias did not differ from non-affected men regarding the majority of the investigated psychosocial outcomes (all *P* > 0.05).	NA	Autism spectrum disorders; Attention deficit hyperactivity disorder; Behavioral/emotional disorders
Cakmak et al. (14) Turkey	Case-control	NA	S: 78 C: 59	Communication, developmental and social-emotional problems	Multivariate logistic regression analysis showed that hypospadias was the independent predictive factor for the problems of the communication and personal-social skills (all *P* < 0.05).	NA	Ages and Stages Questionnaire and ASQ-Social Emotional Scale

*SDS, Self-rating Depression Scale; SAS, Self-rating Anxiety Scale; BDI, Beck Depression Inventory; OR, Odds ratio; CI, Confidence Interval; S, Study group, patients with hypospadias; C, Control group, normal population; NA, Not Available*.

The evidence of psychological disorders in hypospadiac patients reported in the 17 included studies was illustrated in the Discussion Section.

## Discussion

Hypospadias, characterized by an opening of the urethra on the underside of the penis, is one of the most common congenital malformations in boys (26). Based on the published data of hypospadias, most of the studies have been focusing on the surgical techniques and the consequent functional, cosmetic, and sexual outcomes (18, 27, 28), but few studies have concerned the psychiatric symptoms in boys or adolescents with hypospadias. Actually, however, hypospadias alongside with the surgical procedures or surgical outcomes have all been postulated for leading to psychiatric disorders [e.g., depression, anxiety, and timidness; (25, 29)]. However, some studies actually failed to find a positive relationship between hypospadias and psychiatric illness. For example, Mureau et al. conducted a comparative study investigating the psychosocial functioning between patients following hypospadias surgery and the healthy controls (30). The authors compared the differences between the two groups by performing the stratification analysis on the subject age, age at final surgery, penile appearance, the severity of hypospadias, number of operations, and type of surgical procedure, showing that children, adolescents, and adults following hypospadias surgery did not have a higher risk of poor psychosocial functioning as compared to general populations (30). At present, the evidence on the association between hypospadias and the risk of psychosocial disorders remains controversial among different studies.

Though hypospadias is one of the most common malformations, few studies focusing on its psychiatric comorbidity and the correlated data have been conflicting (31). Due to abnormal appearance in the penis with abnormal urination and erection (32), patients with hypospadias were vulnerable to suffering from stress and psychiatric difficulties on account of the negative effects on their social behaviors, school success, gender roles, and self-confidence (14). As illustrated in Table 1, nearly half of the included studies (6/14, 47%) suggested that patients with hypospadias were clearly more liable to suffer from psychosocial disorders than the controls. Berg et al. (11, 12) have conducted two clinical studies to investigate the association between hypospadias and psychosocial illnesses in the 1980s. The authors reported that hypospadias was correlated to more psychiatric symptoms (i.e., hostility, reduction of social relations and emotional relations, anxiety, less self-esteem, and less activity) than the controls in adult age (*P* < 0.05). The mean age of the participants in the study group was 27.2 years, and they received the criterion operation of hypospadias at their age at 3.0–9.5 years (mean: 5.6 years). A study developed in China showed that preoperative hypospadiac patients have significantly higher occurrences of depression and anxiety disorders than the control group (*P* < 0.001) (23). In addition, the authors also found that the postoperative SDS/SAS scores were significantly higher in patients with hypospadias. Furthermore, patients with proximal hypospadias and multiple procedures have a remarkably higher risk of sexual psychiatric problems.

Butwicka et al. (13) have conducted a large sample case-control study which was involving over 470,000 participants (mean age: 13.2 years), they found that the lifetime prevalence of any psychiatric disorders was 9.7% for cases with hypospadias and 7.6% for controls (OR = 1.3, 95% CI: 1.2–1.4). The common psychiatric disorders in hypospadias patients were attention-deficit hyperactivity, autism spectrum, and behavioral/emotional disorders. A previous study showed that boys aged 6–10 years with hypospadias had anxiety than the controls (33). Sandberg et al. had also confirmed the positive relationship between hypospadias and psychiatric morbidities, showing that emotional problems increased with the number of hospital-related experiences (29). Cakmak et al. (14) demonstrated that hypospadias was an independent predictive factor for impairment of communication (odds ratio = 4.06, 95%CI: 1.32–13.37, *P* = 0.015) and personal-social (odds ratio = 5.7, 95%CI: 1.23–26.34, *P* = 0.026) skills impairment. However, a cohort study developed by Ortqvist et al. (18) indicated that psychiatric symptoms (i.e., affective, suicidal, and anxiety symptoms) were more common in hypospadias patients than the healthy controls, but these psychiatric illnesses did not have e markedly difference between the two groups as well as between the severity or phenotype groups. Besides, no statistical significant difference was observed in the four included studies (10, 14, 15, 21). In line with these findings, Mureau et al. (30) demonstrated that psychiatric impairments did not differ obviously between patients with hypospadias surgery and those who underwent surgery for an inguinal hernia. Gender dysphoria and gender identity are two types of psychiatric illnesses. Two eligible studies (20, 34) suggested that patients with hypospadias did not significantly differ from the control subjects with regard to gender identity and gender-role behavior. However, Schönbucher et al. (20) reported that gender-role behavior was remarkably negatively associated with the patients' age at last surgery (*P* < 0.05). Further studies on gender issue in patients with hypospadias are warranted.

According to the above evidence, six included studies (10, 15, 18, 20, 21, 34) explicitly showed that there was a non-significant association between hypospadias and psychiatric illnesses, regardless of whether underwent the surgery or not, while eight included studies (11–14, 16, 19, 23, 24) suggested that patients with hypospadias had a significantly higher risk of psychiatric comorbidity than the controls. Since almost all the patients had received hypospadias surgery, when choosing the operative age, the psychiatric comorbidity should be considered comprehensively to achieve the optimal therapeutic effect. Wang et al. (24) observed a positive association between hypospadias and psychosocial disorders, they subsequently divided the hypospadiac patients into three groups based on age at last surgery, including group A (<10 years), group B (10–18 years), and group C (>18 years). The authors found that the SDS was 47.44 ± 5.88, 53.98 ± 7.23, and 54.25 ± 7.02 in Group A, Group B, and Group C, respectively. On the other hand, the SAS was 44.72 ± 8.44, 49.80 ± 7.25, and 50.66 ± 6.71 in Group A, Group B, and Group C, respectively. According to the statistical analyses, the authors revealed that the SDS, SAS, and the incidence of depression and anxiety differed significantly between Groups A and B (*P* < 0.001), groups A and C (*P* < 0.001), indicating that psychosocial disorders were even more obvious in patients who had completed surgery after 10 years old. However, some investigators indicated that a younger age might be more susceptible to some mental problems. Luo et al. (16) investigated the postoperative behavior changes in children undergoing hypospadias repair surgery and found that the incidence of the negative postoperative behavioral changes (i.e., temper tantrums and emotionality) was 1.5 times higher for children younger than 4 years old as compared to those older than 4 years old. Based on Wang et al. and Luo et al.'s studies, if solely minimizing the “psychiatric burden,” the age for hypospadias repair surgery is recommended from 4 to 10 years.

Whether hypospadias *per se* or surgical procedures have a negative influence on later psychiatric development still needs further investigations. Among the 17 included studies, only Schlomer et al.'s study (19) pointed out that the hypospadias patients were untreated subjects. Notably, regardless of surgery or not, whether hypospadias was associated with psychological disorders was still controversial. Forty-seven percent of the included studies had confirmed the positive association between hypospadias and psychiatric comorbidity, while 35% of the included studies failed to find such an association. Among nine included studies supported this correlation, seven of them reported the hypospadias patients previously underwent surgery, one study reported non-surgery, and other one study did not explicitly the surgery issue. But we could not assess the exact roles of surgical procedures on later psychological development due to none of studies have provided the data of the psychiatric changes in pre- and post-operation.

As we know, this is the first study for summarizing all the evidence of the association between hypospadias and the risk of psychiatric disorders by a comprehensive review. However, several inherent limitations should be taken into account. First, only 17 studies were included for depth profiling. Second, this review could not demonstrate that which type of psychiatric illness is more common in patients with hypospadias due to a wide array of psychiatric disorders that have been described among these included studies. Third, we failed to conduct a meta-analysis due to the limited data, thus how risky psychiatric illness is among hypospadiac patients remains elusive.

## Conclusion

Based on this review, hypospadiac patients' mental illnesses could be commonly detected in their childhood. Boys with hypospadias are recommended to be evaluated on their psychiatric status, which has crucial importance. Besides, proper psychiatric guidance and early interventions from physicians, nurses, and patients may help these children to grow into less affected men.

## Author Contributions

TJ and SZ: project development and data collection. WW and MS: data collection and conceptualization. XL: methodology and investigation. TJ, SL, HF, and SL: original draft and methodology. All authors contributed to the article and approved the submitted version.

## Funding

The Science and Technology Planning Project of Taizhou City, Zhejiang Province (20ywb40), the Social Development Project for the Application of Commonweal Technology of Zhejiang Province (LGF19H050004), the High-level Hospital Construction Research Project of Maoming People's Hospital, and the Natural Science Foundation of Zhejiang Province (No. LQ22H04009).

## Conflict of Interest

The authors declare that the research was conducted in the absence of any commercial or financial relationships that could be construed as a potential conflict of interest.

## Publisher's Note

All claims expressed in this article are solely those of the authors and do not necessarily represent those of their affiliated organizations, or those of the publisher, the editors and the reviewers. Any product that may be evaluated in this article, or claim that may be made by its manufacturer, is not guaranteed or endorsed by the publisher.
